# Zapffe’s Bacteriology

**Published:** 1903-07

**Authors:** 


Zapffe's Bacteriology. A Manual of Bacteriology for Students and Physicians. By Fred. C. Zapffe, M. D., Professor of Histology in the College of Physicians and Surgeons, and Professor of Pathology, Bacteriology and Hygiene in the Illinois Medical College, Chicago. In one 12mo volume of 350 pages, with 150 engravings and 7 full-page colored plates. Cloth, $1.50 net; flexible leather, $2.00 net. Lea's Series of Pocket Text-books, edited by Bern B. Gallaudet, M. D.

Professor Zapffe's compendious manual covers the theoretical and clinical aspects of bacteriology in a manner answering the needs of general practitioners as well as students. The clear and concise manner with which he deals with this subject renders this little volume most valuable. Eliminating unnecessary discussions, he starts at the very beginning, carrying his reader systematically up to the point of gaining a full and comprehensive view, not only of bacteriology, but also of its practical relation to medicine. A course of practical laboratory exercises is likewise included in this comprehensive volume. R. and L.

